# Parameters Influencing Brain Oxygen Measurement by Regional Oxygen Saturation in Postcardiac Arrest Patients with Targeted Temperature Management

**DOI:** 10.1089/ther.2019.0032

**Published:** 2020-02-10

**Authors:** Atsushi Sakurai, Shingo Ihara, Rumi Tagami, Junko Yamaguchi, Atsunori Sugita, Tsukasa Kuwana, Nami Sawada, Satoshi Hori, Tetsuya Taniguch, Kosaku Kinoshita

**Affiliations:** ^1^ Division of Emergency and Critical Care Medicine, Department of Acute Medicine, Nihon University School of Medicine, Tokyo, Japan.; ^2^ Department of Mathematics, Nihon University School of Medicine, Tokyo, Japan.

**Keywords:** regional cerebral oxygen saturation (rSO_2_), postarrest brain injury, cerebrovascular autoregulation, targeted temperature management (TTM)

## Abstract

In several studies, regional cerebral oxygen saturation (rSO_2_) has been measured in patients with postcardiac arrest syndrome (PCAS) to analyze the brain's metabolic status. However, the significance of rSO_2_ in PCAS patients remains unclear. In the present study, we investigated the relationship between rSO_2_ and physiological parameters. Comatose survivors of out-of-hospital PCAS with targeted temperature management (TTM) at 34°C for 24 hours were included. All patients were monitored for their rSO_2_ and additional parameters (arterial oxygen saturation [SaO_2_], hemoglobin [Hb], mean arterial pressure [MAP], arterial carbon dioxide pressure [PaCO_2_], and body temperature]) measured at the start of monitoring and 24 and 48 hours after return of spontaneous circulation (ROSC). Patients were divided into favorable and unfavorable groups, and the correlation between rSO_2_ and these physiological parameters was evaluated by multiple regression analysis. Forty-nine patients were included in the study, with 15 in the favorable group and 34 in the unfavorable group. There was no significant difference in the rSO_2_ value between the two groups at any time point. The multiple regression analysis of the favorable group revealed a moderate correlation between rSO_2_ and SaO_2_, Hb, and PaCO_2_ only at 24 hours (coefficients: 0.482, 0.422, and 0.531, respectively), whereas that of the unfavorable group revealed moderate correlations between rSO_2_ and Hb values at all time points, PaCO_2_ at 24 hours and MAP at 24 and 48 hours. rSO_2_ was moderately correlated to MAP in unfavorable patients. To optimize brain oxygen metabolic balance for PCAS patients with TTM measuring rSO_2_, we suggest total evaluation of each parameters of SaO_2_, Hb, MAP, and PaCO_2_.

## Introduction

In several studies, near-infrared spectroscopy (NIRS) has been used to measure regional cerebral oxygen saturation (rSO_2_) in patients with postcardiac arrest syndrome (PCAS) to predict the outcome and analyze the brain's metabolic status (Meex *et al.*, 2013; Ahn *et al.*, 2014; Storm *et al.*, 2014; Ibrahim *et al.*, 2015). In these studies of PCAS patients in the intensive care unit (ICU), rSO_2_ was significantly lower in nonsurviving (Meex *et al.*, 2013; Ahn *et al.*, 2014) and poor neurological outcome patients (Storm *et al.*, 2014) than in surviving or good neurological outcome patients. In contrast, Ibrahim *et al.* (2015) reported that there was no difference between the rSO_2_ of survivors and nonsurvivors in the ICU setting. Recently, Ihara *et al.* (2019) showed that there was no difference in the rSO_2_ value between severely brain-injured PCAS patients with abnormal amplitude-integrated electroencephalography (aEEG) and mildly brain-injured patients with continuous aEEG wave. This was because the rSO_2_ of patients with abnormal aEEG had a dramatically wider range than that of patients with normal aEEG. It was concluded that this variation in rSO_2_ in patients with severe brain injury may indicate the pathophysiology of postcardiac arrest brain injury owing to impaired cerebrovascular autoregulation (CVAR) and cerebral blood flow (CBF) (Ihara *et al.*, 2019).

Generally, brain oxygenation, measured by rSO_2_, will depend on Fick's principle: rSO_2_ = SaO_2_-CMRO_2_/(1.34 × Hb × CBF) (SaO_2_: arterial oxygen saturation, CMRO_2_: cerebral oxygen metabolic ratio, Hb: hemoglobin). CMRO_2_ changes depending on the body temperature (BT) (Ehrlich *et al.*, 2002) and severity of whole-brain ischemic injury (Tichauer *et al.*, 2009). CBF is described by the Hagen-Poiseuille's equation: CBF = k × ((MAP-ICP) × d^4^)/(L × μ) (k: physical constant, MAP: mean arterial pressure, ICP: intracranial pressure, d: cerebral vessel diameter, μ: blood viscosity, L: length of vessel). Cerebral vessel diameter is regulated by arterial carbon dioxide pressure (PaCO_2_) and μ is influenced by Hb (Nakashima *et al.*, 2017). In the standard ICU setting for PCAS patients, parameters such as SaO_2_, Hb, MAP, BT, and PaCO_2_ are usually evaluated. Therefore, by investigating the relationship between rSO_2_ and these parameters, we could indirectly estimate brain oxygenation in the injured brain. This study is a *post hoc* analysis of our previous investigation (Ihara *et al.*, 2019) estimating the relationship between rSO_2_ and parameters such as SaO_2_, Hb, PaCO_2_, MAP, and BT.

## Methods

This observational study was performed at the intensive care unit at Nihon University Itabashi Hospital. Approval was obtained from the Clinical Research Institutional Review Board (IRB) of the Nihon University School of Medicine Itabashi Hospital (RK-121109-1). Participants of this study were comatose survivors of out-of-hospital PCAS, aged 20 years or older, and treated with targeted-temperature management (TTM) from July 1, 2012 to June 31, 2015. This study included consecutive patients treated with TTM; however, patients were excluded if (1) they died abruptly within 72 hours after cardiac arrest without sufficient evaluation of brain function or (2) they had a history of neurological diseases or brain injury.

The details of the TTM protocol have been described in our previous study (Ihara *et al.*, 2019). In brief, patients who remained comatose after return of spontaneous circulation (ROSC) were treated with TTM at 34°C for 24 hours. After patients with ROSC were sedated (midazolam, 0.08 mg/kg intravenously) and paralyzed (rocuronium bromide, 0.8 mg/kg intravenously) to control shivering, the conditions were maintained with a continuous infusion of midazolam (0.05–0.1 mg/kg/h), fentanyl (1 μg/kg/h), and rocuronium (0.3–0.6 mg/kg/h). The parameters were measured and recorded as follows: mean blood pressure (BP) >65 mmHg, SpO_2_ 94–97%, PaCO_2_ 35–45 mmHg, and Hb <7 g/dL. All patients were monitored for rSO_2_ (INVOS 5100 C; Covidien, Boulder, CO) immediately after the patient's arrival in the ICU. The NIRS probe was placed on the left forehead to detect frontal cerebral oxygen saturation. rSO2 and the other parameters (MAP, SaO_2_, PaCO_2_, and Hb) were evaluated at the start of monitoring and 24 and 48 hours after ROSC. The neurological outcome was assessed using the Cerebral Performance Category (CPC) scale (Cummins *et al.*, 1991) during discharge from the hospital. Patients were divided into two groups according to the outcome: favorable neurological outcome (favorable group) was defined as patients with a CPC score of 1 or 2, and unfavorable outcome (unfavorable group) was defined as patients with a CPC score of 3–5.

Statistical analysis was performed using SPSS (IBM SPSS Version 22). The Fisher's exact probability test, *t* test, and Mann–Whitney U test were used to assess statistical significance of characteristics and parameters between favorable group and unfavorable group. Relationships between rSO_2_ and each parameter were examined with the Spearman's rank correlation coefficient and multiple regression analysis. A *p*-value <0.05 was considered statistically significant.

The ratio of variation to mean value at rSO_2_ and each parameter between favorable groups was evaluated by the coefficient of variation (CV = [standard deviation/mean] × 100). The significant difference in CV between the two groups was calculated using the likelihood ratio test with the hypothesis that k normally distributed populations share the same CV. The web program that we used to statistically analyze CV is www1.fpl.fs.fed.us/covtestk.html. This method was mentioned in a previous study (Ihara *et al.*, 2019). A *p*-value <0.05 was also considered statistically significant.

## Results

The details of patients flow has been described in detail in our previous study (Ihara *et al.*, 2019). In addition, 2 patients died within 72 hours during TTM and they were excluded in the subsequent studies and analysis as was the case in the previous study (Ihara *et al.*, 2019). Forty-nine patients were included in this study, with 15 patients in the favorable group (CPC1: 11 cases, CPC2: 4) and 34 in the unfavorable group (CPC3: 3, CPC4: 11, CPC5: 20). Patient characteristics are presented in Table 1. There were significant differences between the favorable and unfavorable groups with respect to gender male (80% vs. 50%), proportion with shockable rhythm (60% vs 27%), and time from arrest to ROSC (20.9 ± 19.1 vs. 36.4 ± 18.2; minutes). The rSO_2_ value was compared between the two groups, and there was no significant difference at any time point. Hb was significantly larger in the favorable group compared to the unfavorable group at every time point, as was MAP at the start of monitoring and at 24 hours (Table 2).

**Table 1.** Comparison of Characteristics Between Patients in the Favorable and Unfavorable Groups

	Favorable group* n* = 15	Unfavorable group* n* = 34	*p* value
Age, years, mean ± SD	57.8 ± 18.2	67.7 ± 14.2	0.068
Male, *n* (%)	12 (80)	17 (50)	0.049
Cardiac cause, *n* (%)	10 (67)	18 (53)	0.371
Witnessed, *n* (%)	13 (87)	24 (71)	0.228
Shockable rhythm, *n* (%)	9 (60)	9 (27)	0.025
Bystander CPR, *n* (%)	10 (67)	15 (42)	0.146
Time from arrest to ROSC, min, mean ± SD	20.9 ± 19.1	36.4 ± 18.2	0.010
Time from ROSC to rSO_2_ application, min, mean ± SD	351.9 ± 199.4	350.6 ± 177.5	0.845

CPR, cardiopulmonary resuscitation; ROSC, return of spontaneous circulation; rSO_2_, regional cerebral oxygen saturation.

**Table 2.** Comparing of Patient Parameters Between the Favorable and Unfavorable Groups for Every Time Point

	Favorable group* n* = 15	Unfavorable group* n* = 34	*p* value
Start of monitoring			
rSO_2_ (%)	55.5 ± 5.9	57.1 ± 14.0	0.587
SaO_2_ (%)	97.5 ± 2.0	97.1 ± 2.5	0.494
Hb (g/dL)	14.4 ± 2.8	12.1 ± 2.9	0.008
PaCO_2_ (mmHg)	34.9 ± 6.7	35.1 ± 7.8	0.803
MAP (mmHg)	101.2 ± 18.4	87.1 ± 16.6	0.015
Body temperature (°C)	34.3 ± 0.8	33.9 ± 0.6	0.112
After 24 hours			
rSO_2_ (%)	62.9 ± 9.1	57.6 ± 15.8	0.615
SaO_2_ (%)	96.4 ± 3.0	97.0 ± 2.1	0.730
Hb (g/dL)	13.7 ± 3.3	11.7 ± 2.7	0.039
PaCO_2_ (mmHg)	36.5 ± 6.1	36.6 ± 9.4	0.879
MAP (mmHg)	104.3 ± 15.9	85.9 ± 18.2	0.001
Body temperature (°C)	34.5 ± 1.0	34.3 ± 1.0	0.806
After 48 hours			
rSO_2_ (%)	68.9 ± 10.9	64.5 ± 13.3	0.275
SaO_2_ (%)	96.3 ± 2.2	96.1 ± 3.3	0.654
Hb (g/dL)	13.4 ± 3.1	10.8 ± 2.3	0.012
PaCO_2_ (mmHg)	40.0 ± 6.1	37.4 ± 6.9	0.341
MAP (mmHg)	97.5 ± 20.4	85.9 ± 23.0	0.123
Body temperature (°C)	36.3 ± 0.8	35.9 ± 0.8	0.076

Mean ± SD.

rSO_2_, regional cerebral oxygen saturation; SaO_2_, arterial oxygen saturation; Hb, hemoglobin; PaCO_2_, arterial carbon dioxide pressure; MAP, mean arterial pressure.

The CV of the rSO_2_ in the unfavorable group was significantly greater than that in the favorable group at the start of monitoring [CV: unfavorable (24.5) vs. favorable (10.6), *p* = 0.0015] and at 24 hours (27.4 vs. 14.5, *p* = 0.0108) after ROSC. In contrast, there were no significant differences in the CV of rSO_2_ at 48 hours and SaO_2_, Hb, PaCO_2_, MAP, or BT at any time point between the two groups.

The correlation coefficient between rSO_2_ and each parameter at every time point is given in Table 3 for both groups. Table 4 shows the results of the multiple regression analysis between rSO_2_ and each parameter at every time point in the favorable and unfavorable group. In the favorable group, SaO_2_, Hb, and PaCO_2_ had moderate correlations with rSO_2_ at 24 hours, and the standardized partial regression coefficients were 0.482, 0.422, and 0.531, respectively. In the unfavorable group, rSO_2_ was moderately correlated with Hb at all time points, PaCO_2_ at 24 hours and MAP at 24 and 48 hours.

**Table 3.** Correlation Coefficient Between rSO_2_ and Each Parameter at Every Time Point in the Favorable and Unfavorable Groups

	SaO_2_	Hb	PaCO_2_	MAP	BT
Start of monitoring					
Favorable group	−0.397	0.667^*^	0.653^*^	0.428	0.742
Unfavorable group	0.329	0.488^*^	−0.131	0.013	0.107
24 hours after ROSC					
Favorable group	0.409	0.697^*^	0.460	0.421	−0.110
Unfavorable group	−0.026	0.524^*^	0.259	0.558^*^	0.088
48 hours after ROSC					
Favorable group	−0.056	0.621^*^	0.558	0.435	−0.080
Unfavorable group	−0.102	0.622^*^	0.276	0.529^*^	0.485

Correlation coefficient; ^*^ρ < 0.05.

rSO_2_, regional cerebral oxygen saturation; ROSC, return of spontaneous circulation; SaO_2_, arterial oxygen saturation; Hb, hemoglobin; PaCO_2_: arterial carbon dioxide pressure; MAP, mean arterial pressure.

**Table 4.** Multiple Regression Analysis Between rSO_2_ and Each Parameter at Every Time Point in the Favorable and Unfavorable Groups

	SaO_2_	Hb	PaCO_2_	MAP	BT	R^2^
Start of monitoring						
Favorable group	0.119	0.345	0.356	0.123	0.222	0.485
Unfavorable group	0.269	0.579^*^	0.023	−0.150	0.078	0.424
24 hours after ROSC						
Favorable group	0.482^*^	0.422^*^	0.531^*^	0.004	−0.031	0.911
Unfavorable group	0.124	0.555^*^	0.269^*^	0.328^*^	0.223	0.526
48 hours after ROSC						
Favorable group	0.669	0.559	1.052	−0.336	0.003	0.758
Unfavorable group	0.037	0.550^*^	0.205	0.408^*^	0.111	0.647

Standardized partial regression coefficients; ^*^ρ < 0.05.

rSO_2_, regional cerebral oxygen saturation; ROSC, return of spontaneous circulation; SaO_2_, arterial oxygen saturation; Hb, hemoglobin; PaCO_2_, arterial carbon dioxide pressure; MAP, mean arterial pressure; BT, body temperature.

## Discussion

In the present study, there was no significant difference in rSO_2_ values between the two groups at any time point after resuscitation from CA. The value of rSO_2_ in the unfavorable group had a significantly larger variation than that in the favorable group in early stages after ROSC, although there were no significant differences in the variation of the other parameters between the two groups. In the favorable group, SaO_2_, Hb, and PaCO_2_ had moderate correlations with rSO_2_ only at 24 hours. In the unfavorable group, moderate correlations existed between rSO_2_ and Hb at all time points, PaCO_2_ at 24 hours, and MAP at 24 and 48 hours.

In theory, the concentration of Hb must have a binary effect on brain oxygenation. According to Fick's principle, as Hb concentration increases, brain oxygenation will also increase because of the increase in oxygen carrier. On the contrary, based on Hagen–Poiseuille's law, as Hb concentration increases, brain oxygen will decrease as CBF decreases with augmented blood viscosity owing to Hb. However, practically, the relationship between rSO_2_ and Hb concentration obeys Fick's principle, with rSO_2_ having a positive correlation with Hb concentration in cardiac surgical and neurosurgical patients (Yoshitani *et al.*, 2007). In the present study, Hb concentration was significantly positively correlated with rSO_2_ at all time points in the unfavorable group and at 24 hours in the favorable group. This indicates that brain oxygenation based on Hb concentration obeys Fick's principle. Storm *et al.* reported that rSO_2_ was significantly lower in patients with poor outcomes; however, Hb concentration was also significantly lower in those patients (Storm *et al.*, 2014). As they pointed out in their discussion, low rSO_2_ in their study may be due to a low Hb concentration, rather than the severity of brain injury.

In an experimental study of piglets with hypoxia-ischemia, CMRO_2_ decreased after resuscitation (Tichauer *et al.*, 2009). Clinically, Edgren *et al.* (2003) using positron emission tomography study reported that the initial CMRO_2_ of PCAS patients was commonly low, irrespective of whether their outcome was favorable or not. CMRO_2_ depends on body temperature and decreases during hypothermia (Ehrlich *et al.*, 2002), and there was no difference in body temperature between the favorable and unfavorable groups in the present study. Therefore, low CMRO_2_ may not influence the difference in rSO_2_ between groups.

It was reported that in the majority of PCAS patients in their acute phase after cardiac arrest, the CVAR and CBF were either absent or right shifted (Sundgreen *et al.*, 2001). In addition, during the evaluation of rSO_2_ for PCAS patients after ROSC, the impairment of CVAR following CA is associated with poor outcome (Brady *et al.*, 2007; Ameloot *et al.*, 2015; Pham *et al.*, 2015). If CVAR is sustained because of mild brain injury in PCAS patients, rSO_2_ may not change depending on MAP and may change on SaO_2_, Hb, and PaCO_2_ as per the Fick's principle. In this study rSO_2_ moderately correlated with SaO_2_, Hb, and PaCO_2_ and had no correlation with MAP in the favorable group at 24 hours. It indicates that the favorable group may have mild brain injury and CVAR is sustained. Conversely, if PCAS patients with severe brain injury are completely impaired in CVAR and CBF, rSO_2_ and MAP may not be positively correlated. This pattern might explain the patient's situation of the unfavorable group at the start of monitoring. If CVAR is right-shifted, change in CBF depending on MAP and rSO_2_ may positively correlate with MAP. This was observed in patients of the unfavorable group at 24 and 48 hours after ROSC. Ihara *et al.* (2019) reported that the CV of rSO_2_ in PCAS patients with abnormal EEG was significantly larger than those with normal EEG and suggested that it resulted in an impaired CVAR. This hypothesis may not contraindicate the results of this study. Patients with unfavorable outcome should have severe brain injury and the impairment of CVAR. It may indicate wide variation of CBF and wide variation of rSO_2_ in patients of unfavorable group, compared with those of favorable group. There is no standard value of MAP that would be appropriate for all hemodynamic targets (Ameloot *et al.*, 2015). Due to an impaired CVAR, we could control MAP by measuring rSO_2_ to avoid brain ischemia for PCAS management (Sekhon *et al.*, 2016).

The cerebral vasculature constricts by decreasing PaCO_2_, and this CO_2_ reactivity results in a reduction of CBF (Kontos *et al.*, 1977). If CO_2_ reactivity is preserved without brain damage, then rSO_2_ and PaCO_2_ are positively correlated (Booth *et al.*, 2011). In the present study, rSO_2_ and PaCO_2_ were positively correlated in the favorable and unfavorable groups, which suggests that the reactivity of PaCO_2_ was preserved in these patients. On the contrary, there was no significant correlation between rSO_2_ and PaCO_2_ in other conditions. In PCAS patients, CO_2_ reactivity was reported to be preserved, although they had severe brain injury owing to CA (Buunk *et al.*, 1997; Bisschops *et al.*, 2010). As mentioned above, CVAR is impaired in certain brain injuries by CA, and CBF may change depending on MAP. In such a situation, we may not evaluate PaCO_2_ reactivity by this method. We need to measure the response of rSO_2_ value individually in PCAS patients, thus changing PaCO_2_ by controlling the ventilator.

This study, however, has its limitations. If rSO_2_ is measured in patients with a normal or mildly injured brain, rSO_2_ should be correlated with SaO_2_, Hb, and PaCO_2_ and not with MAP. This could explain Fick's formula (Nakashima *et al.*, 2017) and display normal CVAR (Sundgreen *et al.*, 2001; Brady *et al.*, 2007; Pham *et al.*, 2015). In this study, only patients in the favorable group at 24 hours were followed with this hypothesis. Although we could not explain this phenomenon exactly, it may be due to the following reasons: (1) the number of patients may be too small; (2) each parameter was managed within a narrow range, causing the variation range to be narrow; and (3) an unstable brain situation immediately after resuscitation at the start of monitoring and due to change in brain metabolism during the rewarming stage at 48 hours.

Pham *et al.* (2015) reported that early impairment of CVAR following cardiac arrest is independently associated with mortality. Our results suggest that rSO_2_ was moderately correlated with MAP and CVAR and could be impaired in PCAS patients with unfavorable outcome. Ehara *et al*. (2017) showed that in the unfavorable patients, the cerebral rSO_2_ values increased significantly just after the start of ECPR. Taken together, we could predict the outcome for PCAS patients at a very early stage of ROSC by evaluating the relationship between rSO_2_ and blood pressure. Ameloot *et al.* (2015) suggested that there is no “one-size-fits-all” hemodynamic target for the PCAS patients because of impaired CVAR. In this study, rSO2 was changed by MAP, SaO_2_, Hb, and PaCO_2_, depending on patient's brain severity. By evaluation of rSO_2_ in PCAS patients, we could optimize brain oxygen metabolic balance through management of the parameters influencing brain oxygenation such as SaO_2_, Hb, MAP, and PaCO_2_.

## Conclusion

In this study, variation of rSO_2_ was widely dependent on MAP in unfavorable patients, maybe because of impaired CVAR. It will be difficult to predict the outcome by absolute values of rSO_2_ in the case of PCAS patients. To evaluate brain injury in those patients by rSO_2_, we should focus on the relationship between rSO_2_ and blood pressure. Furthermore, in the management of PCAS patients with TTM by rSO_2_, we would suggest total evaluation for each parameter of SaO_2_, Hb, MAP, and PaCO_2_ to optimize brain oxygen metabolic balance.
